# Reconciling Subsistence Emissions and Prevention in Environmentally Sustainable Healthcare

**DOI:** 10.1007/s11673-025-10517-5

**Published:** 2026-07-02

**Authors:** Joshua Parker

**Affiliations:** Lancaster Medical School, Health Innovation One, Bailrigg, Lancaster, LA1 4YW United Kingdom

**Keywords:** Climate change, Distributive justice, Sufficientarinism, Health prevention, Environmental bioethics

## Abstract

With healthcare systems worldwide committing to reduce their greenhouse gas emissions, policymakers must navigate how to reconcile emissions reduction with the delivery of high-quality care. Two main distributive ideals have emerged to guide a just transition to decarbonized healthcare: subsistence emissions and prevention. The subsistence view holds that healthcare emissions that are necessary to meet a threshold of “enough health” are permissible, while emissions exceeding this threshold should be mitigated. The prevention approach, in contrast, prioritizes the most efficient uses of limited emissions through emphasizing measures to prevent the onset of illness and to promote healthy living. Although both frameworks aim to reconcile pursuing the goals of healthcare with reducing the sector’s carbon footprint, their approaches to that end appear to conflict. Subsistence emissions tend to prioritize those below the threshold of enough health, whereas prevention focuses on maintaining health in the already relatively well off. As subsistence emissions offers a compelling strategy for decarbonizing healthcare fairly, the question arises: can it accommodate prevention? This paper argues that these two perspectives can be reconciled by detailing how a subsistence emissions approach can also endorse emissions for measures that prevent individuals from falling below a health threshold.

## Introduction

The urgent need to address climate change has driven healthcare systems worldwide to commit to reducing greenhouse gas emissions (World Health Organization 2024). Mitigation policies aim to either directly reduce greenhouse gas emissions (henceforth “emissions”) or reduce the impact of emissions by enhancing carbon sinks. In some countries, like England, there is a legal requirement to reduce healthcare emissions (National Health Service 2020). A central challenge for policymakers and scholars concerns how healthcare systems can balance their mission to protect health with minimizing the emissions that have traditionally enabled healthcare delivery.

This paper analyses two promising methods of marrying healthcare decarbonization with securing the benefits of modern healthcare. One approach relies on the idea of subsistence emissions (or simply “subsistence”). Subsistence can help demarcate permissible healthcare emissions that are essential to meet basic needs from non-subsistence emissions, which are liable to mitigation (Parker 2025b; Bhopal, Bærøe, and Norheim 2022). Alternatively, healthcare systems can prioritize carbon-effectiveness and drive down emissions by mitigating inefficient uses of healthcare carbon (Bhopal and Norheim 2021). This latter approach tends to emphasize prevention. Despite the appeal of both subsistence and efficiency, they can conflict. Subsistence prioritizes those with the greatest healthcare needs, justifying those emissions that are necessary to fulfil such needs (Shue 1993; Duus-Otterström 2023). In contrast, efficiency-driven prevention tends to benefit those who, in health terms, are comparatively well off. This raises a critical question: should healthcare decarbonization policies favour either subsistence or efficiency, or can these concepts be combined?

In this paper, I defend both concepts as serving a useful purpose in healthcare decarbonization and argue that these approaches can be reconciled by recognizing that subsistence should include prevention measures that secure sufficient health. The paper has three main objectives: (1) Clarifying the concepts of subsistence and prevention in healthcare decarbonization; (2) explaining their potential conflict; and (3) resolving this tension to ensure fair and effective mitigation policies. Despite their central role in healthcare decarbonization, these concepts have received little bioethical analysis. Furthermore, appreciating and addressing the tension between subsistence and prevention is critical for ensuring that healthcare systems are able to decarbonize fairly.

The target of the arguments in this paper is “healthcare”. In this respect, healthcare is to be understood as an organization that serves as a collective agent, formed by the many people who comprise it, which creates a structure through which healthcare goods and services are distributed (Collins 2023). Such goods and services are typically directed towards the efforts of promoting and protecting health, the relief of suffering, the care of the dying, etc. through medical care. In particular, I have publicly funded healthcare systems like the U.K. National Health Service (NHS) in mind, but my arguments are likely to apply to other healthcare organizations as well. It is healthcare as an organization that bears the responsibility for mitigation in this paper.

The argument unfolds in three sections. First, it clarifies subsistence emissions as a sufficientarian concept. Subsistence treats emissions above and below a health threshold differently, permitting only the emissions necessary for a decent level of health, and contrasts this with prevention as a carbon-effective strategy. The second section maps the tension between subsistence emissions and prevention, highlighting how each distributes emissions differently based on what constitutes “enough” health. The final section argues that *securing* sufficient health is essential to sufficientarianism. Since prevention enhances long-term health security, some preventive measures should be included within subsistence emissions. The paper concludes by addressing objections and refining how emissions should be allocated for fair and effective healthcare decarbonization. By integrating subsistence emissions with prevention, this paper offers a framework for balancing the duty of healthcare *qua* organization to protect health with its responsibility to mitigate climate change.

## Subsistence Emissions and Prevention Emissions

This opening section clarifies the concept of subsistence emissions and explains how it is in tension with prevention emissions. Subsistence emissions here are understood within a sufficientarian pattern of distributive justice. Sufficientarians rely on thresholds when distributing goods, and it is how mitigation burdens are allocated around those thresholds that put subsistence emissions in tension with ideals of prevention.

### Subsistence Emissions in Healthcare

Ever since Henry Shue introduced the concepts of luxury and subsistence emissions, they have been an important feature in the climate justice debate (Shue 1993, 2019). Building on the idea of “survival emissions,” Shue argued that not all emissions should be treated equally when it comes to mitigation.^1^ Rather, those emissions that are necessary to fulfil vital needs should be protected from market mechanisms of emissions reduction (Shue 2019). Shue claims that it would be a mistake to “… ignore the fact that some sources [of greenhouse gases] are essential and even urgent for the fulfilment of vital needs and other sources are inessential or even frivolous” (Shue 1993, 55). However, he goes beyond just pointing out that some emissions are necessary to secure more morally urgent ends by also suggesting that this moral urgency should entail a difference in how such emissions are treated in terms of the associated mitigation burdens.

In general, there are constraints on the burdens that agents can reasonably be expected to shoulder in the pursuit of effective mitigation. Subsistence emissions captures this moral principle by claiming that such emissions are morally justified and permissible or even exempt from mitigation responsibilities (Duus-Otterström 2023). Non-subsistence emissions, on the other hand, are unjustified, are therefore liable to mitigation, and ought to be addressed as a priority.^2^ According to Shue, if emissions are necessary to meet basic needs, then they should be spared from mitigation or at least addressed later by mitigation policies (Shue 2019).

Many theorists recognize the sufficientarian spirit of subsistence emissions (Duus-Otterström 2023; Caney 2012; Vanderheiden 2008, 243). Sufficientarianism describes a pattern of distributive justice where emphasis is placed on everyone securing enough of some good(s) (Frankfurt 1982). Sufficientarianism is frequently described as a commitment to two theses (Casal 2007). The first is the “positive thesis.” This states that we have weighty non-instrumental reasons to secure enough of some good(s) (Shields 2012; Timmer 2021a). The positive thesis captures the thought that is it especially morally important that individuals have enough and that those without enough ought to be prioritized. The second thesis is an area where sufficientarians diverge. Some endorse the “negative thesis” (Frankfurt 1982; Axelsen and Nielsen 2016; Crisp 2003). This states that once people have secured enough, no further distributive criteria apply. Others, meanwhile, accept the “shift thesis.” This holds that above a threshold of “enough”, our reasons to benefit change (Shields 2012). The positive thesis is accepted by all sufficientarians, and this is accompanied by either the negative thesis or the shift thesis.

Subsistence emissions is a sufficientarian approach, since it involves adopting both the positive and negative theses. Subsistence emissions endorses the positive thesis by putting significant moral weight on those individuals below the threshold of “enough.” Subsistence emissions recognizes the instrumental value of emissions in securing certain goods. We might disagree on exactly which goods are required to secure enough, but the perspective of subsistence as an approach is clear: it is especially morally important that those without enough are prioritized, and this justifies producing emissions in the pursuit of certain goods. For Shue, there is a basic moral minimum that people are entitled to as a matter of justice, and we have strong moral reasons to benefit those below this threshold. Shue describes this threshold as “the line beneath which no one is to be allowed to sink” (Shue 1980, 18).

Moving to the negative thesis, once individuals have secured enough such that they’ve had their basic needs met or have reached a basic moral minimum, emissions are no longer considered “subsistence.” Above this threshold of “enough,” non-subsistence emissions are unjustified. Hence, subsistence emissions endorses the negative thesis because there is a threshold above which emissions are impermissible, regardless of their purpose. The result is that emissions under the threshold of “enough” are given absolute, or lexical, priority over benefits above the threshold.

It is important to be clear that emissions hold instrumental value and are simply a means to fulfilling certain ends. For subsistence emissions, this end is the sufficientarian concern of ensuring that everyone has enough. The emphasis, then, is on the ends of subsistence and benefitting those below the threshold, rather than the means to achieve this. As Shue points out, the connection between emissions and satisfying basic needs is contingent (Shue 2019). If individuals could access energy for things like transportation and heating without producing greenhouse gases, then the concept of subsistence emissions for energy would be useless. Similarly, if healthcare could be provided without emissions, then subsistence emissions in healthcare would also be redundant.

The question, then, is this: which ends in particular are worthy of the label “subsistence”? This issue is particularly pertinent in healthcare, where there may be a temptation to view all healthcare activities as serving morally salient ends given the value that we tend to place on health. By the same token, it is also important to be careful that any list of non-subsistence medical activities is not cherry-picked or arbitrary.^3^ These considerations speak to the need to have a clear way to distinguish between subsistence and non-subsistence emissions. Ultimately, this depends on the theory of basic needs one adopts (Duus-Otterström 2023). Furthermore, since the sufficientarian threshold of enough is central to the distinction between subsistence and non-subsistence, the practical success of subsistence emissions also depends on this threshold being set appropriately.^4^ How to set the health threshold is discussed in the next section.

A further issue is that, since emissions are contingent on *how* we ensure that everyone has enough, we cannot simply point to the ends which emissions serve and conclude they are *always* justified. Rather, those emissions must be necessary to provide sub-threshold benefits. It hardly seems right to say emissions that are unnecessary to provide even a morally urgent benefit are subsistence. In order to be considered as such, there must be no other opportunities to reduce emissions (Parker 2025a, 241–243). For example, there are broadly two types of inhalers that can be used to treat chronic respiratory diseases like asthma. Metered-dose inhalers produce significant amounts of greenhouse gases, whereas dry-powder inhalers produce fewer (Wilkinson and Woodcock 2022). Both types of inhalers are potentially necessary to secure the benefits of well-controlled asthma, but it is not always the case that metered-dose inhalers are subsistence. This is because dry-powder inhalers can offer similar benefits to metered-dose inhalers but with fewer emissions. It is therefore important to assess the capacities that agents have to minimize their emissions when meeting basic needs when determining which emissions should be considered subsistence (Parker 2022). For emissions to be subsistence they must both be instrumental for sub-threshold benefits and constitute the minimum necessary for agents to satisfy the sufficientarian threshold.

### The Health Threshold

“Subsistence emissions” is a threshold concept, in that it takes a sufficientarian threshold of “enough” and describes how emissions should be allocated in light of that threshold. Below the threshold, emissions are justified when they are essential to produce certain goods and so are not subject to the same mitigation burdens as above-threshold emissions. Consequently, the positive thesis is a necessary but insufficient component of subsistence emissions, as emissions must also be *necessary* to secure certain goods. Those emissions generated above the threshold of enough are unjustified and ought to mitigated. This idea raises two key questions: Where should the threshold be set? And what theory of interests captures the moral concerns underpinning a subsistence emissions approach?

The question of what the relevant currency for sufficientarians ought to be, and where the threshold should be set, are subject to intense debate amongst sufficientarians (Crisp 2003; Benbaji 2005; Casal 2007; Shields 2012; Huseby 2020; Axelsen and Nielsen 2016). Philosophers also propose different thresholds when it comes to subsistence emissions. Shue sets the threshold at “survival or decency” (Shue 1993, 55), whereas Caney is concerned with a “minimal decent standard of living” (Caney 2009, 138). Vanderheiden, meanwhile, opts for a threshold of “basic human functioning” (Vanderheiden 2008, 243). Finally, Roser and Seidel suggest a complex threshold including: “survival … a minimally decent life … and to ensure human dignity” (Roser and Seidel 2017, 144). Whilst there are apparent variations concerning where these scholars place the subsistence threshold, the idea of a decent life features prominently throughout. Clearly there is not the space here to offer a full defence of “a decent life” as the relevant threshold. However, this should have intuitive appeal as something we have reason to value and is clearly a shared threshold amongst numerous philosophers.

This paper is concerned with healthcare, and hence the sufficientarian threshold of interest is a “health threshold” (henceforth simply “the threshold” unless otherwise stated) (Timmer 2024). It is widely accepted that health is a fundamental good that is instrumental to leading a decent life. Whilst there is disagreement over what health is and why it matters morally (Barnes 2023; Schramme 2018), few would doubt that health should feature in an overall package of goods that are necessary for a decent life. Later, a capabilities approach will be adopted as the relevant metric of justice for subsistence emissions. However, it is not necessary at this stage to defend this, so for now I assume that health is the relevant currency for healthcare systems and that resources—specifically those that rely on emissions—are distributed in light of the idea of having “enough health,” if we adopt a subsistence emissions framework (Axelsen and Nielsen 2016; Powers and Faden 2006; Ram-Tiktin 2012, 2016; Timmer 2024; Gustavsson and Juth 2019). I understand “health” in a broad sense, in which health refers to functional states of human bodies that are valuable and important for individuals to have. Threshold views assume that goods can be placed on a continuum (Timmer 2021b). I therefore take it that different health-states can be placed on a continuum and compared between individuals. Health-states on a continuum of health that give rise to claims on healthcare resources are referred to as “healthcare needs” (Schramme 2018; Gustavsson and Juth 2019).

With this in place, we can state the two criteria for subsistence emissions in healthcare.^5^

Subsistence emissions in healthcare are those healthcare emissions that (1) are necessary to secure the health threshold, and (2) for which, at the time of emitting, there is no reasonable alternative way of satisfying the health threshold using fewer emissions.

These two criteria are individually necessary and jointly sufficient to label an instance of healthcare emissions as subsistence. Emissions falling above the sufficientarian threshold, or that are below the sufficiency threshold but are unnecessary or could reasonably be reduced are non-subsistence.

### Subsistence and Mitigation Exemptions

One final clarification regarding the moral status of emissions above and below the health threshold is in order. So far, the claim is that subsistence emissions are justified and therefore permissible, and non-subsistence emissions as unjustified and therefore impermissible. In terms of mitigation responsibilities, as subsistence emissions are permissible, this is often taken to mean that they are exempt from mitigation burdens, unlike non-subsistence emissions.

Duus-Otterström rejects subsistence emissions as being exempt from mitigation burdens (Duus-Otterström 2023). Instead, he argues that even if it is permissible to produce subsistence emissions, what agents must do about these depends on their capacities to compensate for these emissions. As some—for example, the wealthy—have greater resources and opportunities to mitigate or compensate for even those emissions that are necessary to meet their vital needs, an emission’s being permissible does not entail an exemption in all cases. Duus-Otterström therefore accepts the positive thesis, that sub-threshold emissions have absolute priority over above-threshold emissions, as well as the negative thesis that above-threshold emissions are impermissible. However, he rejects the idea that the positive thesis means that emissions are exempt from mitigation and argues that we must ask if below-threshold emissions can be compensated for in some way.

For healthcare systems, then, it is not enough to say that it was necessary to produce emissions to satisfy healthcare needs below the threshold. For these subsistence emissions to be exempt from mitigation, it must also be the case that healthcare systems are unable to mitigate or compensate for these emissions. I contend that a healthcare system is unable to further mitigate its subsistence emissions where doing this would undermine a healthcare system’s ability to meet healthcare needs. Where the resources required to, and costs of, further mitigating subsistence emissions—say, by paying for offsets—would have broader consequences and potentially compromise care, then these emissions are exempt from mitigation burdens.

An example will help illustrate the point about exemptions from mitigation burdens. Imagine there is a medication A that treats an important medical problem like rheumatoid arthritis but that has a substantial carbon footprint. Assume that the healthcare needs associated with rheumatoid arthritis are below a health threshold. As such, the emissions associated with medication A are candidates for being considered subsistence. However, we must also ask if these emissions are the minimum necessary to secure these benefits. If there were a second drug, medication B, which is equally effective, efficient, safe, and well tolerated but has a substantially smaller carbon footprint than medication A, then only medication B would satisfy the requirements of subsistence. However, we could ask whether a healthcare system is able to reduce the emissions from medication B further, perhaps through offsetting. If offsetting was particularly costly or burdensome for a healthcare system such that this limited the ability of others to have similar opportunities for sub-threshold benefits, then this would count in favour of making the emissions from medication B exempt from mitigation burdens.

The concept of subsistence emissions set within a sufficientarian structure around a health threshold provides a useful framework for healthcare systems to reduce their emissions whilst still meeting their goals. Subsistence emissions place special moral importance on helping individuals with healthcare needs below the health threshold. Where emissions are the minimum that are reasonably possible for a healthcare system to produce in order to meet those healthcare needs below the health threshold, they are subsistence. These emissions ought to be treated differently to non-subsistence emissions. Emissions directed towards benefits below the health threshold are non-subsistence if healthcare systems have reasonable opportunities to minimize these without compromising care. Further, due to the negative thesis, emissions associated with above-threshold benefits are non-subsistence and unjustified. With this structure in place, healthcare has a framework for emissions reductions. Non-subsistence emissions are the priority to be eliminated. Emissions that contribute to benefits above the health threshold, and sub-threshold emissions that are unnecessary or can reasonably be reduced, ought to be mitigated. Healthcare’s subsistence emissions should be treated as permissible and exempt from mitigation.

For healthcare systems attempting to drastically cut their emissions, being able to point to some emissions as indispensable in meeting certain healthcare needs is useful. Before moving on to discuss prevention, it is worth highlighting four main advantages of subsistence emissions in healthcare: (a) The concept of subsistence emissions provides a practical framework for addressing healthcare emissions by directing mitigation efforts towards those emissions that are unnecessary or frivolous, and away from those emissions which should be treated differently; (b) by focusing on individuals below a threshold of health, the notion of subsistence emissions tends to shield the worst off from bearing the brunt of mitigation burdens; (c) it provides a degree to which we are justified in treating healthcare as exceptional when it comes to mitigation, (Parker 2025a); and (d) under most schemes of emissions reduction in healthcare, including net zero, some emissions will be very difficult to mitigate and subsistence emissions can explain why these residual emissions are permissible (Sue-Chue-Lam et al. 2024).

## Prevention and Carbon-Effectiveness

Prioritizing prevention, rather than subsistence, offers an alternative way to decarbonize healthcare systems. The logic of prevention is straightforward enough. It takes much more carbon to treat the sick, so if we can prevent people needing carbon-intensive healthcare in the first place, we can both protect health and minimize healthcare emissions. In its outline of how to deliver a net zero healthcare system, the NHS in England state “preventing ill health not only benefits patients but also increases efficiency and reduces emissions” (National Health Service 2020, 39). Bioethicists also endorse prevention within low-carbon healthcare systems. Richie, for instance, argues for “simplicity” in healthcare interventions. This principle involves reducing reliance on medical technology, and one aspect of this is focusing on prevention (Richie 2019, 75–79). Verweij and Ossebaard also note that “in case of interventions with a very large carbon footprint it makes of course sense to look for alternative, more sustainable interventions, for example by prioritising prevention to care” (Verweij and Ossebaard 2024, 69).

The mantra “prevention is better than cure” is at the heart of trends in healthcare over the past fifty years that direct medical resources towards interventions that mitigate risk rather manage symptoms (Rothstein 2008). Prevention refers to strategies intended to ward off illness, disease, and disability, and keep people in reasonable health. Prevention is taken at the population-level by identifying those at risk and acting to mitigate the risk of certain health outcomes materializing. Actions and policies that aim to avoid health problems in the first place are called “primary prevention.” Primary prevention frequently involves interventions outside healthcare, through tackling the social determinants of health (Andermann 2016). Public health strategies that emphasize physical activity, dietary improvement, and minimizing tobacco and alcohol, as well better air quality and so forth, are examples of primary prevention (Romanello et al. 2023). However, some primary prevention does occur within healthcare contexts, with vaccination being a prominent example.

The goal of “secondary prevention” is early detection and management to improve health outcomes. Typically, it is healthcare systems that undertake secondary prevention, particularly in primary care. Identifying and treating those at risk of developing illness—for example, those with dyslipidaemia and hypertension are at risk of cardiovascular disease—are important ways that primary care can contribute to prevention. Chronic disease management can also prevent complications and further health problems. For example, appropriate management of diabetes can ameliorate potential complications like cardiovascular disease, blindness, and kidney problems. Furthermore, screening programmes to detect diseases like cancer early are also examples of secondary prevention.

The treatment of disease and illness, as well as managing symptoms, turns out to be far more carbon-intensive than prevention (Tennison et al. 2021). This is especially true in hospital. Estimates suggest that for inpatients, approximately 105.5 kilograms (kg) of carbon dioxide equivalent (CO_2_e) is produced per patient, per day (Tennison et al. 2021). This comes from all the goods and services required to care for patients in hospital. Pharmaceuticals, staff and patient travel, food, dealing with waste, consumables like syringes, as well as the infrastructure (e.g. buildings) and energy required to deliver healthcare all have a carbon cost (Salas et al. 2020). People rarely stay in hospital for just twenty-four hours, and so these emissions add up over time. Compare the emissions of an inpatient stay to the seventy-six kg of CO_2_e thought to be produced in one outpatient appointment, or estimates that a general practice visit generates sixty-six kg of CO_2_e (Tennison et al. 2021). Of course, outpatient visits will not always involve prevention, but it does help to highlight that inpatient hospital care involves more pollution than community care, generally speaking. If we can prevent patients needing hospital in the first place, or manage their health problems in the community, it is thought there could be big carbon savings.

Prevention policies tend to be carbon-efficient (Richie 2024). They rely on the logic that the best way to ration a scarce and limited resource is through cost-effectiveness analysis. This method is familiar from priority setting in healthcare, but expands the “costs” involved from financial to carbon (Mortimer et al. 2018). Just like with financial cost-effectiveness analysis, what we are looking for is the best ratio of maximal health outcomes for minimal carbon costs. By producing these ratios, we can rank various policies for carbon effectiveness and choose the ones with the most favourable ratios (Bhopal and Norheim 2021). For example, some life cycle assessments have found that emergency caesarean section averts many more disability-adjusted life years than robot-assisted prostatectomy per tonne of carbon (Bhopal and Norheim 2021). On a cost-effectiveness analysis view, then, we should use our limited healthcare carbon budget to perform emergency caesarean sections rather than robot-assisted prostatectomy, assuming the budget precludes doing both.

Small, cumulative benefits across populations means that prevention can maintain health using far smaller volumes of emissions.^6^ Prevention will have very favourable cost-effectiveness ratios because it requires much fewer carbon emissions to keep people well than to treat them once they are sick. For healthcare systems, this is a much more efficient use of emissions because the volume of emissions required to produce a unit of health is much lower. Prevention allows healthcare systems to simultaneously reduce their carbon footprint—by reducing reliance on much more carbon-intensive healthcare—whilst maximizing health outcomes. The idea is that a much more efficient use of emissions in healthcare is to drive down the demand for healthcare in the first place. Healthcare systems can cut their emissions through “lean service” provision whilst also meeting its goals to protect and promote health through preventative strategies (Mortimer et al. 2018).

### The Tension Between Subsistence and Prevention

We can now contrast subsistence, understood as a sufficientarian concept, with prevention, and sketch how these differing decarbonization policies are in tension. Briefly, the tension arises from where the health threshold is set, as well as how benefits above and below the threshold are distributed. Mapping out this tension is critical for appreciating a potential problem in how healthcare systems fulfil their fair share of mitigation burdens. In the next section, a way to address this tension is proposed.

From the proceeding analysis, it should be easy to see how these differing decarbonization policies pull in different directions. In short, subsistence emissions are concerned with benefits below a health threshold and focus on the worst off, whereas prevention attempts to keep people who are above the health threshold there. For instance, subsistence emissions would be concerned with the minimum emissions necessary to treat those who have had a myocardial infarction, whereas medical prevention aims to reduce the risk of a myocardial infarction through management of an individual’s hypertension, dyslipidaemia, and diabetes. A few examples of how these policies work will help illustrate this tension.

Take a lower health threshold. Imagine the health threshold is set such that prevention emissions are above a threshold of “enough.” This is an intuitive place to set the threshold to express the difference between subsistence and prevention. As prevention emissions fail to meet criterion (1) of being subsistence, since they lie above the health threshold, the negative thesis states they are unjustified. Below-threshold emissions are given absolute priority, and so any healthcare emissions must be sub-threshold to be justified. Subsistence emissions rule out prevention emissions because prevention targets those who have enough, and subsistence gives absolute priority to those who do not. With a threshold between subsistence and prevention, it is clear how the negative thesis pulls emissions away from prevention.

The advantage of a low threshold is that it makes the positive thesis easier to justify (Casal 2007). To claim that individuals should not suffer health deprivation below some level and that those individuals should be prioritized makes subsistence emissions appear most compelling. However, the negative thesis makes it harder to justify prevention with a lower threshold. Some may think prevention emissions being ruled out by the negative thesis is not much of a problem if emissions are directed towards those without enough health. Perhaps, in the context of a real need to limit emissions, healthcare emissions should be minimized to those produced by addressing the greatest health needs, marked by being below a threshold of enough.

As much as prioritizing those without enough is of moral importance, I resist the idea that prevention emissions are necessarily unjustified. As I argue further below, there are reasons for sufficientarians to give some weight to prevention, as the latter also helps *secure* the threshold. However, a further reason not to reject prevention emissions as unjustified is that they provide comparatively easy wins in healthcare decarbonization. Low-emissions healthcare is likely to require a small budget of emissions that are necessary to keep people well. A key challenge for sustainable healthcare systems is to simultaneously protect and promote health all whilst minimizing its emissions, and prevention offers one compelling way of doing this. It would therefore be useful if prevention remained part of healthcare’s overall emissions reduction strategy and of a low-emissions healthcare system.

Setting a high threshold, one that includes prevention, offers one way to bring prevention under the auspices of subsistence, making these emissions justified. The advantage of a high threshold is that it makes the negative thesis particularly justified (Casal 2007). When the threshold of “enough” is set sufficiently high to include those who are comparatively healthy and only at risk of becoming unwell without intervention, it is easier to say that any emissions above this are unjustified.

Though setting a high threshold is obviously appealing as a means to justify both subsistence and prevention emissions in healthcare, it does not resolve the tension between prevention and subsistence. To illustrate, consider two principles for distributing under the threshold: “prioritarianism” and “headcount principles.”

Under a higher threshold that includes prevention emissions, prioritarianism gives priority to the worst off. Prevention emissions are directed towards the relatively well off, and so these individuals are likely to be close to the higher threshold. Under a prioritarian distribution of emissions under the threshold, limited emissions are pulled down away from those near the threshold who would benefit from prevention. That is, prioritarianism under the health threshold focuses emissions on something like subsistence since, for the prioritarian, benefits matter more the worse off a person is. Prevention emissions, whilst still being theoretically justified, are unlikely to receive much, if any, of a share of emissions in practice. Being prioritarian under the threshold negates the purpose of raising the threshold, as emissions are moved away from prevention anyway.

Headcount principles, on the other hand, attempt to maximize the number of people who secure “enough” (Shields 2012; Timmer 2021b). Under the threshold, headcounting holds that justice is concerned with *how many* people reach the threshold rather than the size of the benefits to individuals, unlike prioritarian views. The risk involved in headcounting, however, is “excessive upwards transfers” (Shields 2012). A large group who are near the threshold, and who are comparatively well off, would be prioritized over a slightly smaller, very badly off group. Headcounting would therefore prioritize prevention over subsistence and transfer emissions to the relatively well-off compared to the worst off. Subsistence, whilst justified, would receive little emissions on headcounting principles, as prevention uses emissions more efficiently.

Subsistence emissions offer a compelling path to reducing healthcare emissions because they prioritize the worst off using the minimum emissions healthcare can. The negative thesis is a second powerful aspect of subsistence emissions, for it differentiates certain emissions as unjustified. Prevention, on the other hand, is attractive because it utilizes limited emissions to offer healthcare comparatively easy wins in achieving its overarching goal of protecting health. But the negative thesis precludes emissions for prevention. Furthermore, raising the threshold still means choosing between subsistence and prevention because of excessive upwards or downwards transfers, depending on the distributive principle adopted under a higher threshold.

## How to Reconcile Subsistence and Prevention Emissions in Healthcare

Let us now consider how healthcare systems can utilize both subsistence and prevention in policies to reduce their emissions. To reconcile these, I argue that sufficientarians have reason to care about prevention and place some weight on this, as well as by deploying multiple thresholds. The result is that sub-threshold benefits have weighted rather than absolute (lexical) priority, leaving space for the distribution of above-threshold benefits whilst maintaining the ideal of subsistence emissions. As the negative thesis holds particular appeal for emissions reduction, a further threshold above prevention where the negative thesis applies is suggested. Below this second, higher threshold (but above the health threshold), I advocate for the “shift thesis.”

### The Value of Securing Enough Health

To begin, assume again that the health threshold lies between subsistence and prevention. Intuitively, those with cancer, heart failure, and so on can legitimately claim that they are below a health threshold, unlike those subject to initiatives directed at prevention, such as vaccination. Why, then, should subsistence emissions assign weight to above threshold benefits? After all, if subsistence emissions place special moral importance on securing enough, as they have a sufficientarian structure, how can subsistence emissions justify above-threshold benefits?

The core argument can be stated briefly. One reason that sufficientarians might care about above threshold benefits is because of the significant moral weight they place on having enough. As the health threshold has moral importance for sufficientarians, it is not enough that individuals only achieve the threshold. Individuals must be *secure* at or above the threshold and not face material risks that they will fall below the threshold. On this view of sufficientarianism, the negative thesis is rejected, since there is a moral reason to place some weight on above-threshold benefits insofar as they help secure the threshold. Where weight is placed on above-threshold benefits, prevention is of value to the sufficientarian, and some emissions ought to be dedicated to this end.

To build on this sketch, I rely on a capabilities approach as the metric of distributive justice. Different theorists illustrate why securing sufficient health is of moral value by emphasizing differing goods to which health is held to contribute. Some argue that having enough health is necessary for opportunity (Daniels 2007) or that health is valuable because it is a core capability (Nielsen 2015; Venkatapuram 2011; Ruger 2009). Others point to the connection between health and well-being, arguing that having enough health is necessary to avoid suffering (Powers and Faden 2006). Here, I adopt a capabilities approach since, in my view, the idea of secure functioning is key to why sufficientarians should put weight on some above-threshold benefits, and the idea of security has been most well-developed within the context of the capabilities approach (Wolff and de-Shalit 2007, 64–68). However, it is plausible that other theorists will place weight on certain health states being secure.

The capabilities approach is essentially a theory of opportunities. It describes certain conditions or states that allow people to do or be the things they value. Capabilities are, according to Sen, “a set of vectors of functionings, reflecting the person’s freedom to lead one type of life or another” (Sen 1992, 40). “Functionings” describe what individuals can do and be, like being healthy, well-nourished, educated, and so forth. Well-being, on a capabilities approach, is about the functionings that people have based on sets of capabilities that provide opportunities to enjoy such functioning, rather than because they possess certain resources or have their subjective preferences satisfied. Functioning to a certain level is important to lead a decent life, and one must have a sufficient set of capabilities to provide the opportunity for that. To be unhealthy, diseased, and so forth can prevent people pursuing certain life opportunities and consequently their capability to lead a life they have reason to value. Numerous scholars defend health as mattering to justice from a capabilities approach and would view it as an important, if not central, functioning (Nielsen 2015; Venkatapuram 2011; Ruger 2009).

An important distinction present in the capabilities approach is between having the opportunity to achieve a functioning and actually achieving that functioning. Wolff and de-Shalit recognize this distinction, arguing that capabilities should be genuine and secure opportunities to exercise a central functioning (Wolff and de-Shalit 2007, 64–68). What Wolff and de-Shalit draw particular attention to is that one can not only be disadvantaged by the absence of opportunities to exercise central functionings but also if the opportunities they do have are not genuine or are insecure. An opportunity, according to Wolff and de-Shalit, is genuine if one can exercise a functioning without risk to other central functionings and secure if it is reliable going forward. If a functioning is insecure—where there is little realistic prospect of sustaining the function such that people can rely on it—then this too represents a disadvantage.

Take somebody with stage 1 hypertension. Their day-to-day functioning should be unaffected by their blood pressure, as an asymptomatic risk factor. Stage 1 hypertension does not disadvantage in the same way that a problem like chronic obstructive pulmonary disease does. Those suffering the day-to-day symptoms of cough and shortness of breath due to respiratory disease are more obviously disadvantaged by chronic obstructive pulmonary disease than the mild hypertensive. But hypertension is a risk factor for serious problems: heart disease, cerebrovascular disease, renal disease, and so forth. Having hypertension, then, does not necessarily mean that one’s overall health can be relied upon, as one is exposed to risk. To a degree, the health of such a person is at risk and therefore insecure. Since hypertension threatens other important functionings, it is a form of disadvantage.

Stage 1 hypertension is managed through prevention. As an above-threshold benefit, it would not count as subsistence, and if we endorse the negative thesis these emissions are unjustified. However, if what matters is that individuals *secure* the threshold such that not only do individuals achieve the threshold but can rely on staying there, then this provides a reason to place weight on above-threshold benefits. Prevention is important, from the perspective of sufficientarianism, because it contributes to securing the threshold of enough, even if this means providing some above-threshold benefits.

On the account of subsistence emissions provided, however, emissions required to produce above-threshold benefits are precluded by the negative thesis. However, some sufficientarians adopt the shift thesis instead (Shields 2012). Once individuals have secured enough, on the shift thesis, our reasons to benefit them further change. Adopting the shift thesis would still lead us to prioritize below-threshold benefits, though they would now have weighted rather than lexical priority. Further, emissions above and below the threshold are still treated differently when it comes to mitigation. However, on the shift thesis, this becomes a difference of degree, with proportional weighting depending on whether they are above or below the health threshold.

## Objections

Adjusting the concept of substance emissions to include the shift rather than the negative thesis may be seen to go against the spirit of subsistence emissions. When Shue originally proposed subsistence emissions, his concern was with the way that the most economically efficient means for reducing emissions could excessively impact the most disadvantaged:

What if, as is surely in fact the case, some of the sources [of greenhouse gases] that would cost the least to eliminate are essential and reflect needs that are urgent to satisfy, whilst some of the sources that it would cost the most to eliminate are inessential and reflect frivolous whims? (Shue 1993, 55)

Shue’s concern is admittedly put in starker terms than I use here, but the point is clear: the very reason for creating the concept of subsistence emissions was to guard against calculations of cost-effectiveness that result in the worst off shouldering the brunt of mitigation burdens. If the most effective way to decarbonize healthcare is to use carbon more efficiently, and this means maintaining health among the well, one potential consequence is that the worst off pay the most in terms of healthcare mitigation. Combining subsistence emissions with prevention seems to run contrary to subsistence emissions if the point of subsistence emissions is to protect the worst off from calculations of carbon-effectiveness.

It is worth pointing out that subsistence emissions and prevention through cost-effectiveness take somewhat different approaches to minimizing emissions in healthcare. This difference presents an opportunity to marry these approaches without making the worst off pay the most. Subsistence emissions essentially generate a carbon budget around the idea of a health threshold (Parker 2025b). By treating a set of emissions differently in terms of mitigation, healthcare can direct its mitigation efforts towards other non-subsistence sources of greenhouse gas emissions, and that determines the carbon budget for a healthcare system. What I am proposing, then, is that healthcare’s emissions budget should be adjusted to allocate some emissions that are necessary for prevention, given the moral importance of securing the threshold. Both projects can run in parallel, and emissions can be reduced in healthcare not just by focusing on non-subsistence but also through some efficient uses of emissions to prevent ill-health.

Another potential issue is the focus on risk to identify those above the threshold whose health is insecure. Everyone who is above the health threshold will, at some point, be at risk of falling below it. Humans are vulnerable to disease, sickness, disability, and death. To clarify then, for one’s health to be insecure there must be a *reasonable* risk of their health falling below the health threshold. Credible health threats may be because of the size of the risk, or because of how serious the risk is, or because of the way this risk is multiplied by other relevant risks. The person with stage 1 hypertension is at a reasonable risk of having a myocardial infarction if left unchecked. Myocardial infarctions can be fatal, but even those that survive can be left with further cardiac problems, like heart failure. Where the heart cannot function properly because of damage from a myocardial infarction, this seriously disadvantages individuals in terms of their symptoms and functioning. Furthermore, this state also exposes them to risks of other health problems. Hypertension is an insecure health state, then, because it poses a reasonable risk.

One way to help cap an unfettered expansion of the “at risk” category above the health threshold is with the addition of a second, higher threshold as an emissions ceiling. “Reasonably secure health” could denote a second threshold above the lower health threshold set earlier. Where an individuals’ health is relatively secure, as the health risks are small, we can apply the negative thesis that further emissions would be unjustified. Individuals under the threshold of secure health would then have absolute priority over those whose health is reasonably secure. I will not, however, pursue this idea of a second threshold further here.

## Conclusion

This paper has examined the role of both subsistence emissions and prevention in enabling healthcare systems to meet critical objectives, like protecting health, whilst reducing emissions. A key aim was to advance a more systematic understanding of the concept of subsistence emissions in healthcare. Here, subsistence emissions were defended, through a sufficientarian framework, as the minimum emissions that healthcare can reasonably produce to benefit those below the health threshold. While subsistence emissions are an intuitive way for healthcare systems to reduce emissions fairly, they may be in tension with prevention, which offers a promising alternative for emissions reduction.

Both approaches have distinct advantages. Subsistence emissions approaches prioritize the disadvantaged, ensuring that they do not bear disproportionate mitigation burdens, while also providing a practical framework for policymakers. Prevention emissions approaches, meanwhile, offer an efficient means to achieve healthcare goals while reducing emissions. Rather than favouring one over the other, this paper advocates for their integration. Ensuring that individuals secure the health threshold justifies some prevention emissions while reinforcing the need for subsistence emissions. However, subsistence emissions should remain a fundamental exception to mitigation obligations. To help limit the number of prevention emissions, a second threshold that places a ceiling on justified healthcare emissions was suggested. Any emissions above this second threshold are unjustified, as well as non-subsistence emissions other than prevention emissions.

This discussion clarifies subsistence emissions in healthcare and demonstrates their compatibility with prevention. However, further development is needed. Establishing precise, non-arbitrary thresholds is a critical, albeit challenging, task when guiding policymakers. Additionally, while this sufficientarian approach distinguishes permissible from impermissible emissions, it does not address the allocation of emissions within the subsistence budget. Emissions above the health threshold should be allocated based on carbon effectiveness, but the distribution of limited subsistence emissions remains an open question. Addressing these considerations will be essential for implementing subsistence emissions within healthcare policy and practice.

## Data Availability

Not applicable.

## Footnotes

1 Shue credits this concept to Agarwal and Narain. They distinguished between “survival emissions” and “luxury emissions”, though it was of course Shue who developed and popularized the concept by expanding the notion of survival to subsistence (Agarwal and Narain 1991; Shue 2019).

2 The term “non-subsistence” is preferred in this paper over the alternative term “luxury emissions”. This terminological preference is largely because of the topic at hand. A distinction between luxury and subsistence in healthcare seems a little coarse. Moreover, it can be difficult to neatly divide activities into either luxury or subsistence categories. Roser and Seidel contrast the emissions that somebody living in extreme poverty uses to take a bus to secure work with those emitted during an intercontinental flight to attend a wedding. Whilst the former emissions appear to be clearly subsistence, they are less convinced that the second example obviously constitutes non-subsistence emissions. But by the same token, these latter emissions are not uncontroversially subsistence emissions either (Roser and Seidel 2017, 176).

3 Cristina Richie, for instance, lists various treatments of healthcare carbon as inappropriate: fertility treatments,⁠ hormone replacement therapy for menopause,⁠ joint replacement for osteoarthritis,⁠ and other “lifestyle prescriptions” like psychostimulants for attention deficit hyperactivity disorder or medication for lactose intolerance, amongst others.⁠ For Richie, these are clear examples of non-subsistence emissions, as they rely on healthcare *wants*, not healthcare *needs*. However, Richie does not offer a principled way to distinguish healthcare wants from needs. The risk, then, is that any list appears arbitrary without a thoroughgoing way to separate them (Richie 2019, 60–62, 2021).

4 See Axelsen and Nielsen 2016 for a discussion of setting a non-arbitrary threshold, especially regarding health and healthcare.

5 The following closely resembles Göran Duus-Otterström’s helpful clarification of the necessary and sufficient criteria for an instance of greenhouse gas emissions to count as subsistence (Duus-Otterström 2023, 922).

6 One thought is that if societies do much more to address the social determinants of health, then it would be much easier for healthcare systems to decarbonize. States could use policies that address emissions to promote health whilst tackling the social determinants of health. For instance, travel policy could promote “active travel,” which is better for health and simultaneously reduces emissions and air pollution. A comprehensive social policy around decarbonization and health would have a core focus on prevention. Whilst pursuing the co-benefits of policies that reduce emissions whilst also promoting certain goods like health is urgent at a state-level, there is little that healthcare can directly do to address the social determinants of health. For healthcare systems, then, prevention will tend to focus on medical prevention and, given this paper’s focus on the responsibilities of healthcare, I largely consider this rather than broader responsibilities for states.
